# Maladie de Kimura à localisation parotidienne: à propos d'un cas et revue de la literature

**DOI:** 10.11604/pamj.2014.18.294.5093

**Published:** 2014-08-14

**Authors:** Mounir Kettani, Nabil Touihem, Hicham Attifi, Mounir Hmidi, Ali Boukhari, Mohamed Zalagh, Abdelhamid Messary

**Affiliations:** 1 Service d'Orl et Chirurgie Cervico-Faciale, Hôpital Militaire Moulay Ismail, Meknes, Maroc

**Keywords:** Maladie de Kimura, parotide, chirurgie, Kimura disease, parotid, surgery

## Abstract

La maladie de Kimura ou lymphogranulome éosinophile est une pathologie inflammatoire chronique très rare, d’étiologie inconnue. Après avoir considéré que la maladie de Kimura appartenait au groupe des tumeurs de l'endothélium vasculaire et qu'elle pouvait, à ce titre, être assimilée avec l'hyperplasie angiolymphoïde avec éosinophilie chez des patients occidentaux, on pense aujourd'hui qu'il s'agit en réalité d'un processus réactionnel allergique ou autoimmun auquel participent les vaisseaux sanguins, les lymphocytes et les éosinophiles. Nous rapportons un cas de maladie de Kimura à localisation parotidienne chez un Patient de 67 ans qui a consulté devant l'apparition d'une tuméfaction de la région parotidienne droite évoluant depuis deux ans. Le patient a bénéficié d'une parotidectomie total droite et l’étude anatomopathologique de la pièce opératoire est revenue en faveur de la maladie de Kimura. Les suites opératoires été simples. Le recul est d'un an sans récidive.

## Introduction

La maladie de Kimura ou lymphogranulome éosinophile est une affection très rare. Il s'agit d'une pathologie inflammatoire chronique d’étiologie inconnue. Elle touche presque exclusivement les patients originaires d'Extrême Orient, mais peut toutefois survenir de façon plus exceptionnelle chez des sujets caucasiens [1]. Nous rapportons un cas de maladie de Kimura à localisation parotidienne et à travers une revue de la littérature, nous rappelons les principales caractéristiques cliniques, para- cliniques, thérapeutiques et évolutives de cette pathologie.

## Patient et observation

**Figure 1:**
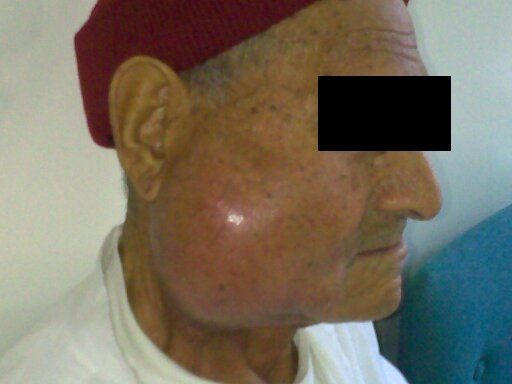
Tuméfaction de la région parotidienne droite

**Figure 2:**
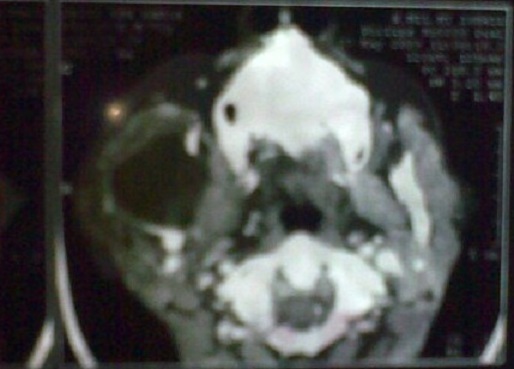
Tomodensitométrie en coupes axiales montrant une glande parotide droite augmentée de taille

**Figure 3:**
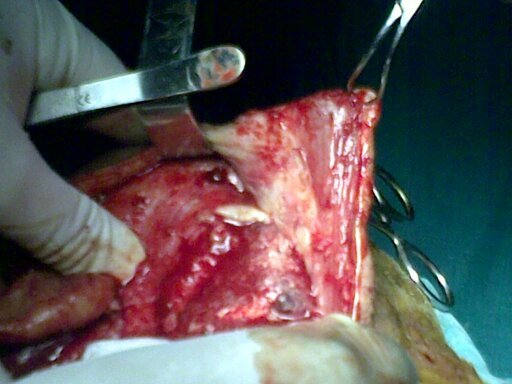
Parotidectomie droite

**Figure 4:**
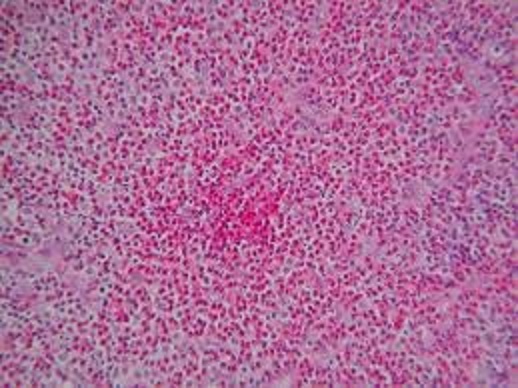
Maladie de Kimura, prolifération vasculaire

Il s'agit d'un patient âgé de 67 ans, diabétique, hypertendu, tabagique chronique 30pa, qui a consulté pour une tuméfaction de la région parotidienne droite, évoluant depuis 2 ans, augmentant progressivement de volume, douloureuse, accompagnées d’épisodes inflammatoires avec absence de trismus et d'asymétrie faciale. L'examen clinique trouvait un patient en bon état général, apyrétique, avec des conjonctives normo colorées. A l'inspection on notait une tuméfaction de la région parotidienne droite avec des signes inflammatoire de la peau en regard (Figure 1). A la palpation, tuméfaction de consistance ferme, mal limitée, douloureuse, non battante, mobile par rapport aux plans superficiel et profond, mesurant environ 6cm de grand axe. La palpation de la région parotidienne controlatérale était sans particularités ainsi que celle des deux régions sous maxillaires. Les aires ganglionnaires cervicales étaient libres. L'examen de la cavité buccale et de l'oropharynx ne montrait pas de pyosialie ni d'hémosialie ni de bombement de la paroi postéro-latérale de l'oropharynx. L'examen ORL complet et l'examen général étaient sans particularités. Une échographie cervico-parotidienne a été demandée et a mis en évidence une glande parotide droite hypertrophiée, d’échostructure hétérogène. Une tomodensitométrie cervico-faciale a montré une glande parotide droite augmentée de taille, de contours irréguliers (Figure 2), infiltrant le tissu graisseux sous cutané en dehors, sans visualisation d'image lithiasique ni de lyse osseuse. Ailleurs, le scanner n'a pas objectivé d'adénopathie cervicale. La cytoponction a révélé une inflammation à cellules polymorphes avec absence de cellules suspectes. Le bilan biologique était normal mis à part une hyperéosinophilie. Cependant la recherche systématique d'une protéinurie est revenue négative. Nous avons décidé de réaliser chez ce patient une paro¬tidectomie total droite avec un examen extemporané (Figure 3). L'exploration chirurgicale a trouvé une glande d'allure inflammatoire. L'examen extemporané était en faveur d'une réaction inflammatoire non spécifique faite essentiellement de lymphocytes, sans signe de malignité. Le patient a bénéficie d'une parotidectomie total droite. Les suites opératoires étaient simples. L’étude anatomopathologique de la pièce a conclue à une maladie de Kimura (Figure 4).

## Discussion

En 1948, le japonais Kimura décrit sous l'appellation « granulome inhabituel associé à des modifications hyperplasiques du tissu lymphoïde » des lésions sous cutanées disséminées accompagnées d'adénopathies et d'une hyperéosinophilie sanguine [2]. La maladie de Kimura affecte principalement les sujets jeunes de sexe masculin [3, 4], avec un sexe ratio variant selon les études de 3 à 7 [5]. Elle touche presque exclusivement les patients originaires d'Extrême Orient, mais peut toutefois survenir de façon plus exceptionnelle chez des sujets caucasiens [1], La maladie de Kimura peut survenir à tout âge, avec un pic de fréquence se situant dans les troisième et quatrième décennies [6]. Il s'agit d'une pathologie inflammatoire chronique d’étiologie inconnue [7]. Après avoir considéré que la maladie de Kimura appartenait au groupe des tumeurs de l'endothélium vasculaire et qu'elle pouvait, à ce titre, être assimilée avec l'hyperplasie angiolymphoïde avec éosinophilie chez des patients occidentaux [8], on pense aujourd'hui qu'il s'agit en réalité d'un processus réactionnel allergique ou autoimmun auquel participent les vaisseaux sanguins, les lymphocytes et les éosinophiles. Il s'agit de nodules ou de placards sous cutanés assez mal limités, de taille variable pouvant atteindre celle d′une paume de main. Leur consistance peut être ferme ou au contraire molle. Les lésions ne sont pas fixées au plan profond, mais adhèrent à la peau sus jacente, qui est le plus souvent de couleur normale ou légèrement brunâtre, exceptionnellement rouge violacée. Elles sont multiples dans 40% des cas, et ont une croissance généralement lente. La localisation préférentielle est cervico-faciale comme l'illustre notre observation (région péri-auriculaire, joues, faces latérales du cou et creux sus-claviculaires) [9, 10]. Les localisations épitrochléenne, axillaire, inguinale et poplitée sont décrites [11]. Les orbites, oreilles, cuir chevelu et avant bras sont rarement atteints. Exceptionnellement les lésions peuvent siéger au niveau des muqueuses [9]. Les atteintes extracutanées sont fréquentes comportent une atteinte des glandes salivaires en particulier la glande parotide; comme le cas de notre patient; et la glande sous maxillaire sous forme d'une augmentation du volume de ces dernières. Les adénopathies locorégionales non inflammatoires sont quasi-constantes. L'atteinte rénale est présente dans 50% des cas et se manifeste par une protéinurie ou un syndrome néphrotique lié souvent à une glomérulonéphrite extramembraneuse [12]. Par ailleurs, les signes généraux sont absents [10].

Sur le plan biologique une hyperéosinophilie sanguine est quasiment retrouvée comme le cas de notre malade et il existe fréquemment une hyperimmunoglobulinémie E. Systématiquement, il faut rechercher une protéinurie qui sera témoin d'un syndrome néphrotique [11, 13]. Les examens radiologiques peuvent être utiles dans l'exploration de la maladie de Kimura, afin de préciser au mieux son extension. Som et Biller [14] ont exploré par scanner et par imagerie par résonance magnétique leur patient atteint d'une maladie de Kimura affectant la glande parotide et les ganglions lymphatiques cervicaux. Le scanner a montré une prise de contraste de la parotide et des ganglions homolatéraux atteints. La prise de contraste des zones atteintes étant similaire à celle de l′artère carotide, la participation vasculaire est ainsi suggérée par les auteurs. L'examen anatomopathologique des lésions montre une hyperplasie du tissu lymphoïde avec des centres germinatifs Florides. Le diagnostic différentiel de la maladie de Kimura se fait principalement avec l'hyperplasie angiofolliculaire avec éosinophilie. Le traitement de la maladie de Kimura n'est pas codifié. Différentes modalités thérapeutiques ont été proposées. Le traitement chirurgical est indiqué en 1ère intention comme c’était le cas de notre patient, il consiste à réaliser une exérèse large et profonde afin d’éviter les récidives qui sont fréquentes [11]. La corticothérapie constitue de loin le traitement médical le plus fréquemment utilisé. Elle est indiquée dans les formes profuses ou inaccessibles à la chirurgie ou systématiquement en cas d'atteinte rénale. Elle est prescrite à la dose initiale de 0,5 à 1 mg/kg/j de prednisone avec une dégression lente sur au moins 6 mois avec une bonne efficacité. Cependant les rechutes sont possibles à l'arrêt du traitement. Une chimiothérapie systémique à base de 5-fluoro-uracil et l'azathioprine a été utilisée en association à une corticothérapie générale mais sans que son utilité soit formellement prouvée [10]. La radiothérapie locale peut être proposé dans les formes réfractaires aux corticoïdes ou lorsque la chirurgie est impossible. La dose totale efficace est comprise entre 25 et 30 Gy.

Actuellement d'autres thérapeutiques sont en cours d'essai sans conclusion formelle. En effet, la Cetirizine a été utilisée et a pu induire chez un patient corticostéroïdes dépendant une rémission complète après 2 mois de traitement [11]. Dans un cas de maladie de Kimura, l'interféron alpha a été prescrit en association à une corticothérapie générale mais n'a pas permis d’éviter une rechute lors de la diminution celle-ci [10]. Le plus souvent, la maladie de Kimura suit une évolution chronique, indolente et bénigne, sans altération de l′état général. Les poussées tumorales peuvent alterner avec des périodes de rémission complète. Les régressions spontanées sont possibles. Les récidives après traitement sont classiques, 15 à 40% [15]. Malgré une présentation clinique inquiétante, avec atteinte des glandes salivaires et présence d'adénopathies, faisant souvent évoquer à tort l′hypothèse de tumeurs des glandes salivaires ou de lymphome, aucune évolution maligne de la maladie n'a jusqu’à présent été signalée. Le pronostic bénin de la maladie doit toute fois être tempéré par la possibilité d'une atteinte rénale parfois associée.

## Conclusion

La maladie de Kimura est une affection rare, qui touche généralement les hommes japonais, âgés de 20 à 40 ans. Elle se caractérise par des nodules sous cutanés prédominant au niveau de la tête et du cou. Son diagnostic est histologique. Le traitement n'est pas codifié mais souvent on a recours à la chirurgie seule comme le cas de notre patient. Cependant, pour certains cas l'indication d'une corticothérapie générale s'avère nécessaire. Le pronostic de la maladie reste bon.
